# Links between Human LINE-1 Retrotransposons and Hepatitis Virus-Related Hepatocellular Carcinoma

**DOI:** 10.3389/fchem.2016.00021

**Published:** 2016-05-11

**Authors:** Tomoyuki Honda

**Affiliations:** ^1^Department of Viral Oncology, Institute for Virus Research, Kyoto UniversityKyoto, Japan; ^2^Division of Virology, Department of Microbiology and Immunology, Osaka University Graduate School of MedicineSuita, Japan

**Keywords:** L1, retrotransposon, hepatitis C virus (HCV), hepatitis B virus (HBV), hepatocellular carcinoma

## Abstract

Hepatocellular carcinoma (HCC) accounts for approximately 80% of liver cancers, the third most frequent cause of cancer mortality. The most prevalent risk factors for HCC are infections by hepatitis B or hepatitis C virus. Findings suggest that hepatitis virus-related HCC might be a cancer in which LINE-1 retrotransposon, often termed L1, activity plays a potential role. Firstly, hepatitis viruses can suppress host defense factors that also control L1 mobilization. Secondly, many recent studies also have indicated that hypomethylation of L1 affects the prognosis of HCC patients. Thirdly, endogenous L1 retrotransposition was demonstrated to activate oncogenic pathways in HCC. Fourthly, several L1 chimeric transcripts with host or viral genes are found in hepatitis virus-related HCC. Such lines of evidence suggest a linkage between L1 retrotransposons and hepatitis virus-related HCC. Here, I briefly summarize current understandings of the association between hepatitis virus-related HCC and L1. Then, I discuss potential mechanisms of how hepatitis viruses drive the development of HCC via L1 retrotransposons. An increased understanding of the contribution of L1 to hepatitis virus-related HCC may provide unique insights related to the development of novel therapeutics for this disease.

## Introduction

Liver cancer, 80% of which is hepatocellular carcinoma (HCC), accounts for 9% of all cancer deaths worldwide (Jemal et al., 2011; Tateishi and Omata, 2012). The major causative agents of HCC are hepatitis viruses, such as hepatitis B virus (HBV) or hepatitis C virus (HCV) (Jemal et al., 2011; Tateishi and Omata, 2012). HBV belongs to the *Hepadnaviridae* family, which has a relaxed circular DNA (rcDNA) as a viral genome (Beck and Nassal, 2007; Nguyen et al., 2008). HCV belongs to the *Flaviviridae* family, which has a nonsegmented, positive-stranded RNA as a viral genome (Hijikata et al., 1991; Grakoui et al., 1993; Aly et al., 2012). Both viruses cause chronic infections, with approximately 350 and 170 million people worldwide affected by chronic HBV and HCV infections, respectively (Parkin, 2006; Aly et al., 2012). It is now clear that chronic HBV and HCV infections play critical roles in the development of HCC (Jemal et al., 2011; Forner et al., 2012; Tateishi and Omata, 2012). However, the precise mechanisms of hepatocarcinogenesis in chronic hepatitis virus infections are still unclear.

Long interspersed nuclear element-1 (LINE-1 or L1) retrotransposons are genetic elements that constitute approximately 17% of the human genome (Lander et al., 2001). Because most L1s are 5′ truncated, most of them are defective, while 80–100 copies are still retrotransposition-competent and utilize a “copy-and-paste” mechanism to retrotranspose to new genomic loci (Brouha et al., 2003; Beck et al., 2010). Aberrantly expressed or dysregulated L1s are considered a major source of endogenous mutagenesis in humans (Levin and Moran, 2011; Burns and Boeke, 2012). L1 retrotransposition occurs in germ cells, pluripotent stem cells, at early stages of human embryonic development (van den Hurk et al., 2007; Beck et al., 2011; Levin and Moran, 2011; Klawitter et al., 2016) and in somatic cells, such as neuronal progenitor cells or cancer cells (Muotri et al., 2005; Iskow et al., 2010). Many epidemiological studies suggest a linkage between L1 and cancers (Shukla et al., 2013; Rodić et al., 2014; Harada et al., 2015). However, in most cases, it is unclear whether L1s are activated in normal cells before clonal expansion or in cancer cells at the later stage of carcinogenesis (Goodier, 2014).

Among cancers, hepatitis virus-related HCC is considered to be a cancer in which L1 might be involved (Shukla et al., 2013). Firstly, by far the majority of L1 *de novo* insertions detected in cancer tissues has been found in cancers of epithelial origin (Goodier, 2014). Secondly, HBV and HCV have a potential to suppress host defense mechanisms that can also control L1 retrotransposition (Gale and Foy, 2005; Chang et al., 2012; Yu et al., 2015). Thirdly, endogenous L1 retrotransposition was demonstrated to activate oncogenic pathways in HCC. Fourthly, several L1 chimeric transcripts with host or viral genes are found in hepatitis virus-related HCC (Lau et al., 2014). Here, I will summarize potential linkages between hepatitis virus-related HCC and L1s. Firstly, I will review how HBV could affect L1 retrotransposon activity. I will then introduce current understandings of the relationship between HCV and L1. Finally, I will discuss possible L1-mediated mechanisms that may induce HCC. Understandings of possible links between virus-related HCC and L1 may open a new avenue for the development of novel therapeutics for this disease.

## A potential link between HBV and L1 in HCC

**Figure 1:**
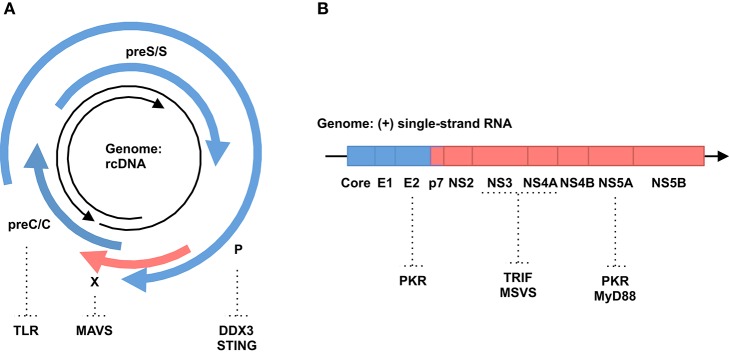
**Genomic structure of hepatitis viruses and immune invasion by their proteins. (A)** HBV. Black arrows indicate HBV genomic rcDNA (from 5′ to 3′). Blue and red arrows indicate structural and non-structural proteins, respectively. Host genes in immune responses targeted by HBV proteins are shown. **(B)** HCV. Black arrow indicates HCV genomic RNA (from 5′ to 3′). Blue and red boxes indicate structural and non-structural proteins, respectively. Host genes in immune responses targeted by HCV proteins are shown.

The 3.2-kb HBV genome encodes four, partly overlapping open reading frames (ORFs): preC/C (core and Hepatitis B e-Antigen [HBeAg]), P (viral polymerase), preS/S (Hepatitis B surface Antigen [HBsAg]) and X (non-structural protein [HBx]) genes (Figure 1A). In the nucleus, the genome is converted into covalently closed circular DNA (cccDNA). From this cccDNA, all viral RNAs, including pregenomic RNA (pgRNA) as a replication intermediate and viral mRNAs, are transcribed. Viral proteins such as core and polymerase proteins and pgRNAs are assembled into the nucleocapsid within the cytoplasm. In the nucleocapsid, pgRNA is reverse transcribed into rcDNA. All these HBV-related nucleic acids have the potential to trigger innate immune responses in infected cells (Ait-Goughoulte et al., 2010). If these immune responses cannot clear HBV, the virus establishes a chronic infection, which is known to increase the risk of developing liver cirrhosis and HCC (Gonzalez and Keeffe, 2011).

Type I interferons (IFNs) play a major role in anti-viral immunity (Katze et al., 2002). Association of IFNs with IFN receptors activates JAK1 and signal transducer and activator of transcription 1/2 (STAT1/2). Then, these proteins interact with interferon regulatory factor 9 (IRF9) and form a potent transcription factor, upregulating the expression of several hundreds of IFN-stimulated genes (ISGs). These ISGs suppress viral replication and spread through various mechanisms described elsewhere (Katze et al., 2002). IFN is used to control HBV replication, indicating that IFN is a restriction factor (Dienstag, 2008). For example, tetherin, an IFN-inducible transmembrane protein, inhibits HBV virion secretion (Yan et al., 2015). Zinc finger antiviral protein (ZAP) is upregulated in IFN-treated cells and restricts HBV replication through downregulation of pgRNA (Mao et al., 2013). On the other hand, HBV has a variety of strategies to counteract IFN signaling (Figure 1A). HBsAg, HBeAg and HBV virions inhibit Toll-like receptor (TLR)-mediated antiviral responses (Visvanathan et al., 2007; Wu et al., 2009; Vincent et al., 2011; Woltman et al., 2011). HBV polymerase suppresses IRF3 activation by interacting with the host RNA helicase, DDX3 (Wang and Ryu, 2010; Yu et al., 2010). HBV polymerase also disrupts ubiquitination of the stimulator of interferon genes (STING) and blocks innate immune responses against cytoplasmic DNA (Liu et al., 2015). Expression of HBx protein inhibits virus-induced expression of the IFN gene by promoting the decay of mitochondrial antiviral signaling protein (MAVS) (Wei et al., 2010; Kumar et al., 2011). Furthermore, HBV abrogates IFN signal transduction by impairing either STAT1 nuclear import or phosphorylation (Christen et al., 2006, 2007; Lütgehetmann et al., 2011). All the listed mechanisms that suppress IFN signaling could also activate L1 retrotransposon, because IFN has been shown to inhibit the expression and retrotransposition of L1 (Yu et al., 2015). The mechanisms underlying the inhibitory effect of IFN on L1 remain unclear. However, MOV10 is an attractive candidate to mediate this inhibitory effect, because MOV10 is an IFN-inducible gene and suppresses L1 retrotransposition (Schoggins et al., 2011; Goodier et al., 2012). Collectively, immune suppression by HBV may activate the expression and retrotransposition of L1 elements.

In addition, HBV may also modulate L1 expression epigenetically. L1 retrotransposition activity is usually suppressed in most somatic cells by host DNA methyltransferase-mediated DNA methylation of its promoter (Ishizu et al., 2012; Castro-Diaz et al., 2014). In cancer cells, global DNA hypomethylation occurs at various genomic loci including those containing DNA repeats and/or retrotransposons (Ehrlich, 2002; Hatziapostolou and Iliopoulos, 2011). Many studies have reported hypomethylation of the L1 loci in HCC and HBV infection (Shitani et al., 2012; Zhang C. et al., 2013; Gao et al., 2014; Zhu et al., 2014). In particular, L1 hypomethylation is likely to be linked to poor outcomes of HCC (Gao et al., 2014; Zhu et al., 2014). Given global hypomethylation occurs in the host genome (including the L1 loci) during HBV infection, this may upregulate L1 expression, potentially removing an obstacle to L1 transposition in liver cells. In addition, some chimeric transcripts, such as HBx-L1, are detected in HCC and associated with a poor prognosis, further supporting the link between HBV-related HCC and L1 (Lau et al., 2014).

## A potential link between HCV and L1 in HCC

The 9.6-kb HCV genome contains a single ORF, encoding a polyprotein precursor of approximately 3000 amino acids. The polyprotein is cleaved by host and viral proteases, producing structural (core, E1 and E2) and non-structural (P7, NS2, NS3, NS4A, NS4B, NS5A and NS5B) proteins (Figure 1B). The replication of HCV starts with the synthesis of a full-length, negative-stranded RNA intermediate, which in turn works as a template for the *de novo* production of positive-stranded genomic RNA. Thus, HCV replicates without a known DNA intermediate stage. HCV genomic RNA is highly structured and contains double-stranded regions in various portions (Tuplin et al., 2002; Zhang S. et al., 2013). Double-stranded RNAs (dsRNAs) are also generated during the replication cycle of HCV. Such dsRNAs are potent inducers of innate immune responses, mainly through TLR3 and retinoic acid inducible gene-I (RIG-I) signal pathways (Li et al., 2012). However, the immune responses induced by HCV are not strong enough to eradicate the virus (Battaglia and Hagmeyer, 2000).

Although it is thought that non-retroviral RNA viruses are not integrated into host genomic DNA, we and others have demonstrated that they do become integrated into the host genome via host retrotransposon machineries (Geuking et al., 2009; Horie et al., 2010). Likewise, HCV cDNA is reportedly detected in patients infected with HCV (Zemer et al., 2008). Because the involvement of HIV was ruled out in all the HCV cDNA-positive patients, it is hypothesized that host retrotransposons might be involved (Zemer et al., 2008). However, the retrotransposons responsible for this phenomenon remain unidentified and the involvement of retroviruses other than HIV is not ruled out. Because the 3'UTR of HCV is not polyadenylated, the contribution of L1, whose substrates are usually polyadenylated, to this phenomenon seems to be unlikely. However, several reports propose alternative retrotransposition mechanisms by L1, termed internal priming or twin priming, where a poly-A tail is not required to prime reverse transcription (Ostertag and Kazazian, 2001; Srikanta et al., 2009). These alternative mechanisms may explain how HCV RNA could be reverse transcribed by L1, despite lacking a poly-A tail. It is also unknown whether HCV cDNA is integrated into the host genome or exists as extrachromosomal DNA. A recent report showed that fragments homologous to HCV genes are present in the rabbit and hare genomes, which might suggest the possibility that cDNA of an HCV ancestor has been integrated into the host genome (Silva et al., 2012). These observations imply that some linkages between HCV and retrotransposon activity might exist.

Most HCV-infected patients develop a chronic infection, suggesting that HCV has developed successful strategies to evade host immune responses (Gale and Foy, 2005) (Figure 1B). For instance, the HCV NS3/4A protease cleaves the Toll/IL-1 receptor domain-containing adaptor inducing IFN-β (TRIF) adaptor protein and MAVS to impair TLR3 and retinoic acid-inducible gene-I (RIG-I) signaling pathways, respectively (Foy et al., 2005; Li K. et al., 2005; Li X.-D. et al., 2005). NS5A and E2 proteins suppress the signaling of the interferon-dependent induced protein kinase R (PKR), a key molecule in the innate immune system (Gale et al., 1997; Taylor et al., 1999). The interferon sensitivity-determining region (ISDR) in the NS5A protein interacts with the death domain of myeloid differentiation primary response 88 protein (MyD88), a major adaptor protein in TLR signaling, and impairs its signaling (Abe et al., 2007). All these mechanisms that suppress IFN responses against HCV could in turn activate retrotransposons, such as L1, in infected cells, because IFN and IFN-inducible genes, such as MOV10, are shown to suppress retrotransposition of L1s as described above (Schoggins et al., 2011; Goodier et al., 2012; Yu et al., 2015).

The HCV core protein has an oncogenic potential (Moriya et al., 1998; Shimotohno, 2000). One mechanism put forward for this is that the core protein modulates host gene expression pathways which may activate oncogene expression (Shrivastava et al., 1998; Marusawa et al., 1999; Shimotohno, 2000; Watashi et al., 2001; Ray et al., 2002). In addition to the core protein, NS5A protein also stimulates NF-κB signaling (Ray et al., 1995; Gong et al., 2001; Park et al., 2002; Waris et al., 2003). Similarly, HCV proteins may stimulate the expression of L1 retrotransposons. Indeed, the infectious HCV virion reportedly activates HIV long terminal repeats (LTR) and upregulates gene transcription (Sengupta et al., 2013). However, studies investigating whether HCV proteins have the potential to stimulate L1 expression and/or retrotransposition have not been reported so far.

## Possible mechanisms of L1 involvement in HCC development

**Figure 2:**
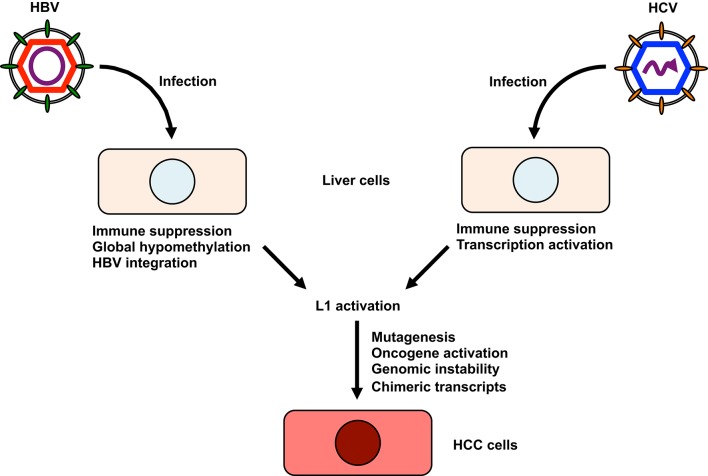
**The possible involvement of L1 in the development of hepatitis virus-related HCC**. HBV infection leads to global hypomethylation of genomic DNA and suppresses host immune responses. HBV also integrates its sequence into the host genome, altering the host transcriptome. HCV can activate transcription of host genes and suppress host immune responses. Furthermore, HCV cDNA might be formed in HCV-infected cells. I hypothesize that these changes could be associated with L1 retrotransposition. L1 activation may lead to the mutagenesis of host tumor suppressor genes, the activation of oncogenes, host genomic instability, and the production of new chimeric transcripts or activation of cytidine deaminases, all of which contribute to the development of HCC.

Although a definitive role for L1 activity in contributing to HCC etiology has not been established thus far, investigating a possible link between L1 activation and the development of HCC would be of considerable interest for a number of reasons (Figure 2). Firstly, L1s, when aberrantly expressed or dysregulated, can be major sources of endogenous mutagenesis in humans as described above (Levin and Moran, 2011; Burns and Boeke, 2012). Any potential disruption of tumor suppressor genes by L1 retrotransposition could contribute to the development of HCC. Indeed, L1 was shown to be a crucial source of mutations that can reduce the tumor-suppressive capacity of somatic cells (Shukla et al., 2013). A subset of L1 *de novo* insertions identified in cancer tissue occurred at genes commonly mutated in cancer (Lee et al., 2012). Secondly, L1 *de novo* insertions can affect the expression of nearby genes and the genes in which they inserted (Lee et al., 2012; Shukla et al., 2013). Intragenic L1 insertions usually coincide with reduced gene expression (Lee et al., 2012). For example, L1 insertions into the tumor suppressor mutated in colorectal cancer (MCC) gene coincides with its downregulation (Shukla et al., 2013). MCC is expressed in liver and suppresses the oncogenic β-catenin/Wnt signaling pathway frequently activated in HCC (Fukuyama et al., 2008). If an L1 insertion occurs close to an oncogene, L1 could enhance oncogene expression, resulting in the development of HCC. For example, the telomerase reverse transcriptase (TERT) gene is one of the most common genes associated with L1 *de novo* insertion (Ding et al., 2012; Lau et al., 2014). Since aberrant expression of TERT is associated with tumor development, L1 insertion near the TERT locus may have a role in carcinogenesis (Cohen et al., 2007; Nault et al., 2015). L1 insertion at the transcriptional repressor suppression of tumorigenicity 18 (ST18) gene activates its expression (Shukla et al., 2013). ST18 is a candidate oncogene in liver, because the expression of ST18 is upregulated in several liver cancer cells and in tumors in a mouse-model for inflammation-driven HCC (Shukla et al., 2013). Thirdly, L1 provides sites that could lead to genomic rearrangements (Burwinkel and Kilimann, 1998). Such genomic rearrangements contribute to genomic instability (Burwinkel and Kilimann, 1998; Ehrlich, 2002). Fourthly, L1 retrotransposition could contribute to new splice donor or acceptor sites, which could alter the host transcriptome and might enhance HCC progression (Singer et al., 2010). Lastly, L1 retrotransposition occasionally creates new chimeric transcripts, which might enhance the progression to HCC. An example of this mechanism is the L1-MET transcript, a chimeric transcript that consists of the c-MET oncogene and an intronic L1 sequences (Zhu et al., 2014). The expression of L1-MET has been shown to be correlated with that of c-MET (Zhu et al., 2014). Because L1-MET is associated with poor prognosis in cancer (Wolff et al., 2010; Hur et al., 2014), L1-MET might be associated with a poor prognosis for HCC via the activation of c-MET signaling (Zhu et al., 2014).

Taken together, I conclude two potential roles for L1 elements in the development of hepatitis virus-related HCC. The first relates to a chimeric transcript specific to HBV-related HCC, HBx-L1, which can be detected in more than 20% of HBV-related HCC and correlates with a poor outcome (Lau et al., 2014). The promoter of the HBx gene transcribes HBx-L1 from the locus that is normally silent in the genome. Knockdown of HBx-L1 reduces migratory and invasive properties of HBV-positive HCC cells. HBx-L1 overexpression confers growth advantage and promotes cell migration and invasion regardless of its chimeric protein-coding potential, suggesting that HBx-L1 is a long non-coding RNA that promotes HCC phenotypes. Furthermore, it has been shown that HBx-L1 affects β-catenin/Wnt signaling, a major pathway in the oncogenesis of HBV-related HCC, confirming its role in HCC (Whittaker et al., 2010; Lau et al., 2014). In addition to this, I hypothesize the other possible role of L1 as a potent inducer of the expression of cytidine deaminases, such as activation-induced cytidine deaminase (AID) and apolipoprotein B mRNA editing enzyme, catalytic polypeptide 3 (APOBEC3). Since transgenic mice expressing AID genes invariably induce tumors, this suggests that cytidine deaminases may have a carcinogenic potential (Okazaki et al., 2003; Takai et al., 2009). APOBEC3 is a protein family of seven proteins in human: APOBEC3A, B, C, DE, F, G, and H (Schumann et al., 2010; Vieira and Soares, 2013). Members of the APOBEC3 protein family restrict replication of not only retroviruses such as HIV, but also retrotransposons, HBV and HCV (Harris and Liddament, 2004; Vieira and Soares, 2013). Among APOBEC3 proteins, APOBEC3G seems to have a major role in HIV restriction (Chaipan et al., 2013; Vieira and Soares, 2013). APOBEC3G also has the restriction activity against LTR retrotransposons in the mouse genome (Esnault et al., 2005; Schumacher et al., 2008). All members of the human APOBEC3 protein family of cytidine deaminases restrict L1 retrotransposition with APOBEC3A, B, C and F having the strongest inhibitory effect (Muckenfuss et al., 2006; Kinomoto et al., 2007). For HBV and HCV, APOBEC3G is a major restriction factor (Vartanian et al., 2010; Peng et al., 2011; Kitamura et al., 2013). HBV and HCV somehow stimulate the expression of cytidine deaminases (Vartanian et al., 2010). Furthermore, L1 activation reportedly increases the expression of the mouse APOBEC3 gene in mouse embryonic fibroblasts (Yu et al., 2015). Taken together, hepatitis viruses, directly and/or maybe indirectly via L1 activation, induce the expression of cytidine deaminases, which may hyperedit host genomes, resulting in the accumulation of deleterious mutations in the genome and the development of HCC (Okazaki et al., 2003; Takai et al., 2009; Vartanian et al., 2010).

## Conclusion and perspective

Presented lines of evidence suggest potential links between hepatitis virus infection and L1 retrotransposon activity. Especially, L1 hypomethylation or some L1 chimeric transcripts are associated with poor prognosis of HCC, suggesting that it can have a significant effect on HCC phenotypes and supporting the idea that HCC is a cancer in which L1 plays a role. However, knowledge of how L1 activation by chronic hepatitis virus infection enhances the development of HCC is still limiting. Further accumulation of examples of recurrent L1 insertion sites in the host genome or recurrent chimeric transcripts specific to hepatitis virus-related HCC will be promising ways to understand L1 involvement in HCC etiology. Single cell analyses of L1 retrotransposition events and expression in tumor cells and surrounding normal cells may enhance these processes. Understanding the potential roles of L1 in HCC may open avenues to developing novel therapeutics, such as RNA interference against HCC-specific L1 chimeric transcripts.

## Author contributions

TH wrote the manuscript and approved it for publication.

### Conflict of interest statement

The author declares that the research was conducted in the absence of any commercial or financial relationships that could be construed as a potential conflict of interest.
